# Clinical impact of L1CAM expression measured on the transcriptome level in ovarian cancer

**DOI:** 10.18632/oncotarget.9291

**Published:** 2016-05-11

**Authors:** Samira Abdel Azim, Michaela Duggan-Peer, Susanne Sprung, Daniel Reimer, Heidi Fiegl, Afschin Soleiman, Christian Marth, Alain G. Zeimet

**Affiliations:** ^1^ Department of Obstetrics and Gynecology, Medical University of Innsbruck, 6020 Innsbruck, Austria; ^2^ Department of Obstetrics and Gynecology, Laboratory for Clinical Biochemistry, Medical University of Innsbruck, 6020 Innsbruck, Austria

**Keywords:** ovarian cancer, L1CAM, targeted therapy, RT-PCR, survival

## Abstract

**Background:**

High expression of L1 cell adhesion molecules (L1CAM) has been repeatedly shown to be associated with aggressive disease behavior, which translates in poor clinical outcome in various cancer entities. However, in ovarian cancer results based either on immunohistochemistry or cytosolic protein quantifications remained conflicting regarding clinical behavior. In the present work we assessed L1CAM expression on the transcriptome level with the highly sensitive quantitative real-time PCR (qRT-PCR) to define its relevance in ovarian cancer biology.

**Results:**

There was a significant difference in L1CAM high and low mRNA expressing cancers with regard to disease-free (p=0.002) and overall survival (p=0.008). L1CAM proofed to be an independent predictor for disease progression (HR 1.8, p=0.01) and overall survival (HR 1.6, p=0.04). Furthermore, a significant positive correlation between the level of L1CAM and the grade of tumor differentiation (p=0.04), the FIGO stage (p=0.025) as well as the histological subtype (p= 0.002) was found.

**Methods:**

This study included fresh frozen tissue samples of 138 patients with FIGO I-IV stage ovarian cancer. L1CAM mRNA expression was determined using qRT-PCR. In the calculations special attention was put on the various histological subtypes. In survival analysis median L1CAM mRNA expression obtained in the entire cohort of ovarian cancer samples was used as a cut-off to distinguish between high and low L1CAM mRNA expression.

**Conclusion:**

L1CAM mRNA expression appears to play a substantial role in the pathophysiology of ovarian cancer that is translated into poor clinical outcome. Additionally humanized L1CAM antibodies, which can serve as potential future treatment options are under testing.

## INTRODUCTION

Ovarian cancer is the fifth most common cancer in females and due to the obscure symptoms mostly diagnosed in advanced stage [1]. The latest research has led to a different classification system in epithelial ovarian cancers, according to their differences in genesis, molecular background and behavior. Currently type I and type II ovarian cancers are distinguished [2]. Type I ovarian cancers include the low-grade serous, low grade endometrioid, clear cell as well as mucinous carcinomas. It is the understanding that low-grade ovarian cancers are slow growing, genetically stable tumors with a low mitotic index that arise in or from precursor lesion such as borderline serous ovarian tumors hence they are referred to as LGSC (low-grade serous ovarian cancers). At the time of diagnosis often the ovaries are solely affected and they most commonly show mutations in the BRAF, PTEN and KRAS gene [3]. Type I ovarian cancers are known to respond poorly to platinum based chemotherapy, however the 10-year-survival rates generally exceed those of the prototype of type II tumors.

Type II ovarian cancers incorporate the high-grade serous cancers, carcinosarcomas and undifferentiated carcinomas, which are diagnosed at a late stage and usually have a poor prognosis [4]. They grow rapidly, have a high mitotic index and are genomically unstable with frequent mutations in p53. Furthermore, type II cancers develop more frequently on a background of deficient homologous recombination repair e.g. germline or somatic BRCA 1/2 mutations [5]. There is growing body of evidence to show that type II ovarian cancers evolve from the fallopian tube and are generally susceptible to platinum based chemotherapy regimens.

At present the classical treatment of debulking surgery followed by platinum based chemotherapy is the same for both types of ovarian cancer. About 50% of patients achieve complete remission after this treatment, however, the 5-year survival rate remains under 40% [6]. In fact 25% of ovarian cancers are *a priori* platinum resistant, and 75% of patients with platinum sensitive tumors relapse within the first 2 years of diagnosis [7]. Cancers relapsing at an interval longer than 6 months after completion of a platinum-based chemotherapy are considered to be platinum sensitive and can be reinitiated by platinum containing drugs. Unfortunately, after a certain time nearly all cancers develop platinum resistance.

As platinum drugs represent the most essential backbone in systemic ovarian cancer treatment, it is of utmost importance to uncover the molecular mechanisms leading to platinum resistance. This will be crucial to truly improve the clinical outcome of that disease with an unacceptable rate of mortality.

L1CAM (CD171) is a cell adhesion molecule that belongs to the immunoglobulin (Ig) supergene family and is a transmembrane glycoprotein of 200–220 kDa. L1CAM is involved in cell migration and axon guidance during neurogenesis [8–10]. The gene of L1CAM is located on the X-chromosome (band Xq28) and comprises of 29 exons of which 28 are coding [11]. L1CAM can be cleaved from the cell surface by the metalloproteinase ADAM10. This shedding of the ectodomain results in the release of the soluble L1CAM (sL1CAM) of about 200kDA and the membrane bound form (mL1CAM) [12, 13]. Outside of neuronal tissue L1CAM expression was found to be associated with various human malignant tumors [14] such as pancreatic tumors, colon cancer, melanoma, renal cell and endometrial carcinoma and was linked to a poor prognosis [15–18]. In ovarian cancer L1CAM expression was previously studied by immunohistochemistry (IHC) on paraffin-embedded samples [19–22] and by enzyme-linked-immunosorbant assay (ELISA) as well in lysates of serous ovarian cancers as in the corresponding ascitic fluid [23, 24]. However, in contrast to other tumor entities results remained conflicting in ovarian cancer. We have included a summary of all relevant published studies on L1CAM expression and ovarian cancer with the main results (see Supplementary Table S1).

Therefore this study for the first time intended to investigate the clinical relevance of L1CAM determined on the transcriptome level by an alternative method, namely the quantitative real-time polymerase chain reaction (RT-PCR) in ovarian cancer.

## RESULTS

A total number of 138 ovarian cancer samples and 32 healthy ovarian tissue samples were analyzed for L1CAM mRNA expression. For included cancer patients the median observation period was 44.0 months (range 1–242 months). The clinicopathologic characteristics of the patient collective are listed in Table 1.

**Table 1:** Clinicopathologic characteristics of included patients

Variable		L1CAM mRNA expression values (arbitrary units)		
		Number (per cent) n=138	Median (Min-Max)	p-Value
FIGO stage **)	I	27 (20%)	0.06 (0.00-3.43)	**0.01**
	II	11(8%)	0.16 (0.01-3.18)	
	III	76 (55%)	0.24 (0.00-3.21)	
	IV	24 (17%)	0.50 (0.01-7.37)	
Grade **)	G1	6(4%)	0.90 (0.01-0.27)	**0.01**
	G2	78 (58%)	0.19 (0.00-4.11)	
	G3	52 (38%)	0.39 (0.00-7.37)	
	unknown	2(1%)		
Histology **)	serous	69 (50%)	0.38 (0.00-4.11)	**<0.01**
	mucinous	44 (32%)	0.13 (0.00-3.43)	
	endometrioid	25 (18%)	0.20 (0.01-7.37)	
Residual disease *)	macroscopically tumor-free vs.	55 (40%)	0.19 (0.00-7.37)	0.14
	any tumor residual	83 (60%)	0.27 (0.00-4.11)	
Platinum sensitivity **)	no recurrence	50 (36%)	0.11 (0.00-3.43)	**<0.01**
	refractory	12 (9%)	0.32 (0.00-7.37)	
	resistant	21 (15%)	0.24 (0.01-4.11)	
	sensitive	54 (39%)	0.43 (0.00-3.21)	
	unknown	1 (1%)		
Recurrence *)	no	50 (36%)	0.11 (0.00-3.43)	<0.01
	yes	88 (64%)	0.34 (0.00-7.37)	
Age (years) *)	< 62.8	69 (50%)	0.23 (0.01-3.43)	0.76
	> 62.8	69 (50%)	0.24 (0.00-7.37)	
Type *)	I (low grade serous carcinoma	36 (26%)	0.12 (0.00-3.43)	0.01
	II (high grade carcinoma)	102 (74%)	0.34 (0.00-7.37)	
TP53 mutation *)	no	23 (17%)	0.10 (0.00-1.61)	**0.01**
	yes	46 (33%)	0.32 (0.00-2.98)	
	unknown	69 (50%)		

*) calculated using Mann-Whitney-U

**) calculated using Kruskal-Wallis.

**Figure 1:**
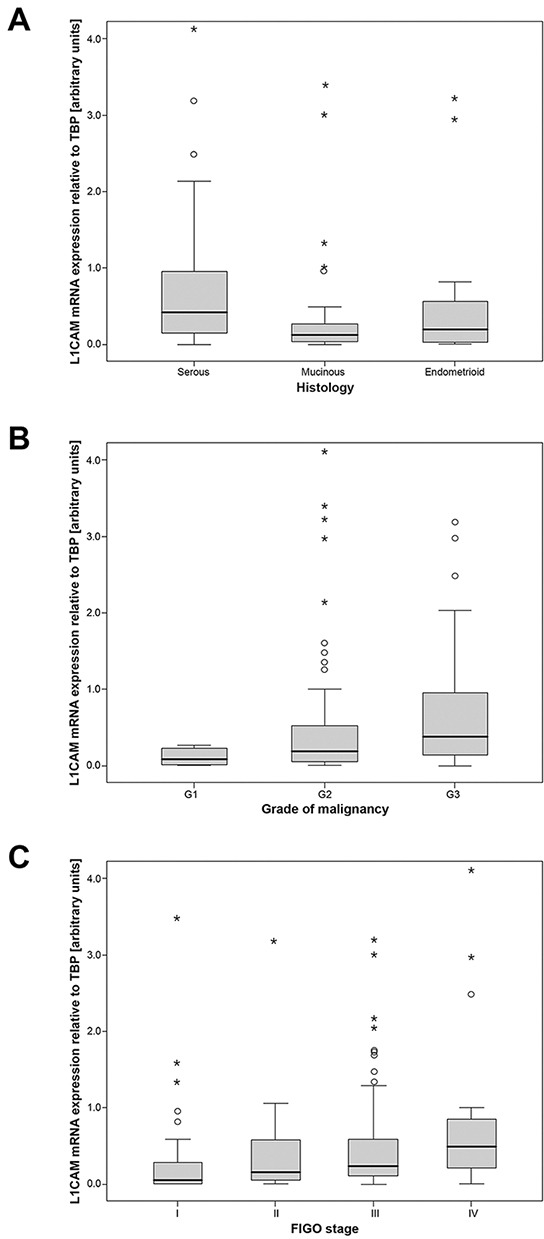
L1CAM mRNA expression in ovarian cancer tissues L1CAM mRNA expression stratified according to **A.** histologic subtypes **B.** tumor grade **C.** tumor stage. Relative quantitations of L1CAM and TBP (housekeeping gene) mRNA expression were assigned by comparison with a standard curve that was generated by serial dilutions of RNA from HTB-77 carcinoma cell line. All samples were analyzed in triplicates. Mean ratios of L1CAM/TBP relative quantitations expressed as arbitrary units are shown.

The L1CAM mRNA expression in the malignant tissue was significantly higher than in the normal healthy ovarian tissue. Median L1CAM mRNA concentration in cancers was 0.23 (L1CAM expression relative to TBP as arbitrary units). In normal ovarian tissues it was 7.2-fold lower compared to malignant tissues (p<0.001). There was a significant difference in L1CAM expression according to various histological subtypes, with the highest expression in serous ovarian cancer and the lowest in mucinous tumors (p=0.003) (Figure 1). High L1CAM expression was associated with high tumor grade (p=0.04) and L1CAM mRNA levels increased with tumor stage (p=0.025) (Figure 1).

There was no significant difference in L1CAM mRNA expression according to the patients' age (median age: 62.8 years).

In 68% (n=94) of the cases p53 status was known. In 67% of these cases p53 was mutated. The mRNA expression of L1CAM was 3.6-fold higher in the p53 mutated carcinomas as compared with cancers displaying no p53 mutation (p=0.004).

Furthermore, according to histopathologic features the distinction between type I and type II ovarian cancer was possible. L1CAM mRNA expression in type I cancers was 2.9-fold lower than in type II tumors (p=0.01).

The L1CAM mRNA expression in ovarian cancer also differed according to platinum response during first line chemotherapy. Interestingly the L1CAM expression was 1.5-fold higher in those women with platinum refractory cancers compared to those being sensitive (0.32 vs 0.22). Patients who were platinum resistant had median L1CAM levels of 0.24. Analysis of the subgroup of patients who did not experience recurrence in the median observation period of 105 months (8.7 years) revealed a 2.9-fold lower L1CAM mRNA expression in comparison to those who recurred at any time during or after chemotherapy.

In the subgroup of patients without macroscopic residual disease after primary debulking surgery (n=55) L1CAM mRNA levels were 2.7-fold higher in those who developed resistant disease as compared with those who either never relapsed within follow-up period or those who experienced platinum sensitive recurrences. However, this difference in L1CAM expression did not reach statistical significance.

**Figure 2:**
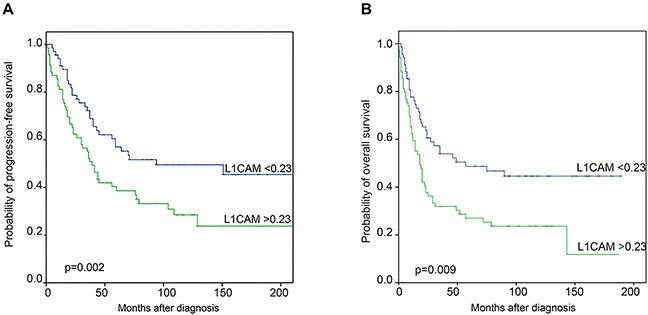
Kaplan Meier survival analysis and L1CAM mRNA expression **A.** Progression-free and **B.** Overall survival in 138 ovarian cancer patients according to the median L1CAM mRNA expression as cut-off value.

Univariate survival analysis of all 138 ovarian cancer patients using the median L1CAM mRNA expression as cut-off revealed that high levels of L1CAM had an adverse prognostic impact as well for progression-free (PFS) (p=0.002) as for overall survival (OS) (p=0.009). Kaplan Meier curves are shown in Figure 2A and 2B. The median PFS was 33 months and 18 months for patients with low and high L1CAM expressing cancers, respectively. The median overall survival was 59 months as compared with 39 months in patients with low and high L1CAM mRNA expressing tumors, respectively.

Furthermore, we compared the L1CAM mRNA expression in the tissue of patients, who after debulking surgery had no residual macroscopic disease with those who had residual disease. Our cohort included 55 (40%) patients without macroscopic residual disease and 83 (60%) with residual disease of any diameter. Between both groups no significant difference in L1CAM expression could be revealed (p=0.12).

In multivariate analyses high L1CAM tissue mRNA levels were found to be an independent predictor for disease progression (HR 1.8; p=0.01). Additionally, residual disease (HR 1.9; p=0.03) proved to be an independent factor for PFS as shown in Table 2a. In multivariate overall survival analysis L1CAM expression (HR: 1.6; p=0.04), FIGO stage (HR: 2.9; p=0.01), age (HR 2.0; p<0.01) and platinum sensitivity (HR: 7.9; p<0.01) were found to be independent predictors for overall survival (see Table 2b).

**Table 2a:** Multivariate analysis of progression-free survival

Factor	p-value	HR	95% Confidence Interval
L1CAM (< median vs. > median)	**0.01**	**1.8**	**1.1 – 2.7**
FIGO stage (I/II vs. III/IV)	0.10	1.9	0.9 – 3.9
Age (< median vs. > median)	0.17	1.3	0.9 – 2.0
Residual disease (macroscopically tumorfree vs. any tumor residuals)	**0.03**	**1.9**	**1.1 – 3.4**
Platinum Sensitivity (sensitive vs. refractory/resistant)	**n.d.**	**n.d.**	

**Table 2b:** Multivariate analysis of overall survival

Factor	p-value	HR	95% Confidence Interval
L1CAM (< median vs. > median)	**0.04**	**1.6**	**1.0 – 2.5**
FIGO stage (I/II vs. III/IV)	**0.01**	**2.9**	**1.3 – 6.4**
Age (< median vs. > median)	**<0.01**	**2.0**	**1.3 – 3.2**
Residual disease (macroscopically tumorfree vs. any tumor residuals)	0.50	1.2	0.7 – 2.3
Platinum Sensitivity (sensitive vs. refractory/resistant)	**<0.01**	**7.9**	**4.6 – 13.6**

**Figure 3:**
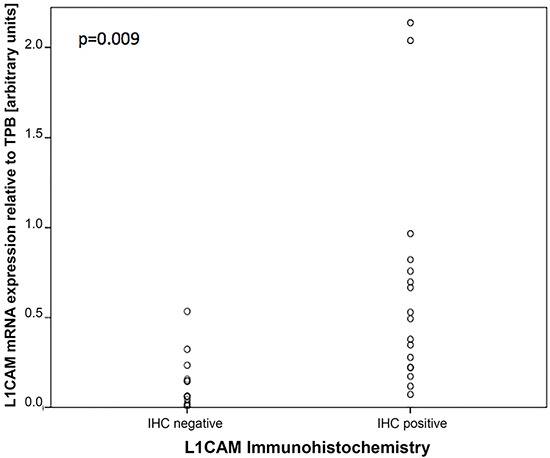
Correlation between L1CAM expression on IHC and absolute L1CAM mRNA levels

Additionally, an immunohistochemcial (IHC) analysis for L1CAM expression on paraffin-embedded slides was performed for a subset of our cohort of patients. On IHC 58% of tumors were L1CAM positive and 42% were negative. The median mRNA value of L1CAM in this subset of patients was 0.23 which was the same as for the whole cohort. Comparing the IHC and RT-PCR results a significant correlation was found between IHC and L1CAM expression on mRNA level (p=0.009) (see Figure 3).

## DISCUSSION

L1CAM expression is found in many different human malignancies and has been shown to be linked to a bad prognosis. Its expression in ovarian cancer has previously been studied using immunohistochemistry [19–22] where the presence of L1CAM was associated with an adverse clinical outcome. Furthermore, the L1CAM expression was analyzed using a quantitative ELISA in ascitic fluid of serous ovarian carcinoma. This study demonstrated a correlation of the shed soluble L1CAM with a decreased progression-free and overall survival. The same study also analyzed the full length membrane bound form of L1CAM in cancer lysates, which correlated with an increased risk of residual disease after debulking and was higher in type-II carcinomas [23].

This is the first study where the L1CAM expression was measured on a transcriptional level by real-time PCR in human ovarian cancer with the aim of a more precise and reproducible quantification of L1CAM activity.

We found a significant correlation of the L1CAM mRNA expression in ovarian cancer with FIGO stage and tumor grading. Furthermore, the L1CAM mRNA expression was significantly higher in those patients who experienced recurrent disease during the observational period.

The p53 mutated ovarian cancers, which most are type II ovarian cancers, had a significantly higher L1CAM mRNA expression compared to the p53 wild type. Crucial effects of p53 are mediated by miRNAs, such as miR-34a [25]. Previous work has shown that miR-34a levels are up-regulated in p53 wild type cell lines of various human tumors. Contrariwise p53 mutant cell lines do not exhibit this up-regulation of miR-34a [26]. In endometrial cancer an inverse correlation between miR-34a and L1CAM expression was found. Especially clear cell and serous endometrial cancers had very low miR-34a levels indicating a loss of miR-34a expression [27]. Correspondingly, high L1CAM transcript levels were most frequently found in serous followed by endometrioid ovarian carcinomas and the lowest L1CAM mRNA was detected in mucinous subtype.

Usually peritoneal metastatic lesions of ovarian cancers are superficial and can easily be removed by peritonectomy. However, from previous work we know that L1CAM plays a crucial role in epithelial-mesenchymal-transformation (EMT) [28] and that this transformation towards the mesenchymal phenotype of peritoneal ovarian carcinoma cells may facilitate their invasion into deeper tissue layers and may thus delimitate surgical resectability of the disease. This hypothesis has been established on the basis of the findings of Bondong et al. who reported a direct relationship between residual disease after primary debulking surgery and levels of L1CAM protein as determined by ELISA from ovarian cancer lysates. However, on the transcriptome level we were not able to confirm the association between the levels of tumor L1CAM mRNA and the magnitude of residual disease after radical surgery. Although it has been shown that residual disease is highly dependent from tumor biology [29], it should nonetheless be taken into account that other factors, like the surgeon's skills, the hospital size, the patient's performance status etc. may also affect this variable significantly.

L1CAM levels were inversely associated with platinum resistance. Lowest levels were found in sensitive tumors, higher L1CAM expression in resistant and highest levels in refractory tumors. This confirms published data that up-regulation of L1CAM within cancer cells assists chemoresistance. Results of previous work in human glioblastoma cells showed a reduced apoptotic response after treatment with chemotherapeutic drugs in glioblastoma cells. An increased L1CAM expression lead to a lower expression of caspase-8 and thereby increased apoptotic resistance of tumor cells [30].

Accordingly, the high L1CAM mRNA expression measured by RT-PCR significantly worsened progression-free (p=0.002) and overall survival (p=0.008) in univariate analysis. This could also be confirmed in the multivariate survival analysis for both PFS and OS. So far most of the results revealing L1CAM expression as an adverse prognostic factor in various cancer entities have been obtained with IHC. However, in ovarian cancer available IHC data on L1CAM are very poor. Regarding clinical outcome, the mentioned ELISA-determined L1CAM protein levels from tissue lysates failed to show a prognostic significance in serous ovarian cancer. In contrast, soluble levels of L1CAM determined out of the ascites of the same patients, proved to be a marker for poor progression-free survival and chemoresistance [23]. As no head to head comparisons between the methods for measuring L1CAM expression is available, it is difficult to classify reliably RT-PCR. Nevertheless in this cohort L1CAM mRNA expression was an independent prognostic marker for PFS and OS almost doubling the relative risk for recurrence and death. We found that L1CAM negative patients on IHC staining also had low mRNA L1CAM levels. However, regarding positive results there was a higher percentage of L1CAM positivity in IHC analyses compared to RT-PCR. This finding is in line with previous results that IHC may result in an higher percentage of positivity due to the described uneven distribution of L1CAM-expressing small cell clusters [18, 25]. In so far one substantial weakness of whole tissue qRT-PCR compared to IHC, is that L1CAM mRNA expression in those small tumor cell clusters in otherwise L1CAM negative cancers can be underestimated. However, it has been shown that these small islands of L1CAM positive cells in cancers are of clinical relevance [18]. In addition limitations of retrospective analyses must be considered as a further weakness of the presented study.

Moreover, in 2012 Schäfer et al. demonstrated in an *in vivo* murine model that L1CAM could be a suitable target for treatment of ovarian cancers when L1CAM-specific humanized antibodies were combined with conventional chemotherapy, e.g. paclitaxel. They found that blockage of L1CAM led to an increase in apoptosis and a decrease in tumor vascularization, caused by a down regulation of VEGF expression [31]. Therefore, a therapy with humanized anti-L1CAM antibodies in addition to standard chemotherapy regimens could potentially positively affect patients' clinical outcome.

A recent study on antibody therapy to human L1CAM in pancreatic carcinoma cells in a transgenic mouse model showed also a significant reduction of tumor size after treatment with the mAb L1-9.3/2a. However, antibody treated tumor cells showed significantly increased levels of EGF, which may induce EMT [32]. This finding could indicate that L1CAM directed antibody treatment could also have pro-tumorigenic effects by promoting tumor progression and metastasis [33]. As this study was carried out exclusively on pancreatic carcinoma cells it remains completely unclear whether these unfavorable effects could prove also true for ovarian cancer.

The herein presented data reveal that measurement of L1CAM expression at the transcriptome levels in ovarian cancer tissue could potentially serve as a tool to predict the clinical outcome in ovarian cancer and underscores the importance of this adhesion molecule in the tumor biology of ovarian cancer. Especially with regard to the possible treatment option with humanized L1CAM antibodies, head to head comparisons are however needed to define the most reliable method to determine the L1CAM status in ovarian cancers.

## MATERIALS AND METHODS

### Specimens and patients

Tissue samples of 138 patients treated between 1989 and 2000 at the Department of Obstetrics and Gynecology, Medical University of Innsbruck were retrospectively analyzed in this study. Clinical tumor stage was assessed according to the FIGO classification. Clinical data including tumor stage, overall survival, follow-up and recurrent disease were extracted from the hospital database and patients' records. Tissue samples were randomly selected. All carcinoma patients underwent primary debulking surgery (except for one individual due to impaired performance status) followed by a platinum-based chemotherapy. Patients in FIGO stage I were only included with either FIGO stage Ic tumors or FIGO stage Ia, Ib high-grade (G3) tumors.

Platinum resistance was defined as relapse within 6 months after the end of first-line therapy. Platinum refractory was defined as disease progression under platinum based chemotherapy.

The study was approved by the Ethics Committee of Medical University of Innsbruck (reference number UN2014-0301).

### Quantitative determination of L1CAM levels

#### RNA extraction and reverse transcription

Tumor specimens were obtained immediately after surgery and brought to our pathologist, who prepared a nearly 100% tumor cell containing part of the tissue which was pulverized under cooling with liquid nitrogen and stored at −80°C. Total cellular RNA was extracted from the tumor specimens using the TRI reagent (Sigma-Aldrich, Seelze, Germany) according to manufacturer's instructions. Integrity was evaluated by assessing the 18S- and 28S-ribosomal RNA bands in 2% ethidium bromide-stained agarose gel. RNA concentration was measured by spectrophotometric analysis.

Reverse transcription of RNA was performed in a final volume of 20 ml containing 1x RT-Buffer (50mM Tris-HCl, pH 8.3, 75mM KCl, 5mM MgCl2), 40U of rRNasins RNase Inhibitor (Promega, Madison, USA), 10mM dithiothreitol, 200U of M-MLV Reverse Transcriptase (Life Technologies, Carlsbad, CA, USA), 5 mM random hexamers (Life Technologies, Carlsbad, CA, USA) and 400 ng of total RNA. The samples were first incubated at 65°C for 5 min and then quick-chilled on ice. After adding the M-MLV enzyme, the samples were incubated at 25°C for 10 min and at 37°C for 50 min, followed by a period of 15 min at 70°C to inactivate the reverse transcriptase enzyme.

#### Primers and probes

Primers and probes for the TATA box-binding protein (TBP; a component of the DNA-binding protein complex TFIID as an endogenous RNA control) were used according to Bieche et al. [34] Primers and probes for L1CAM were determined with the assistance of the computer program Primer Express (Life Technologies, Carlsbad, CA, USA). BLASTN searches were conducted to confirm the total gene specificity of the nucleotide sequences chosen for the primers and probes. To prevent amplification of contaminating genomic DNA, the probe was placed at the junction between exon 12 and exon 13. L1CAM forward primer: 5′-TTC GTC CTG AAG CAC TGT TGT C-3′; L1CAM reverse-primer: 5′-GGA GCG CCT GTG CCC-3′; L1CAM TaqMan probe: 5′-FAM-ATC CTC GTC CAG CCA CTG AAC A-3′-TAM.

#### Real-time PCR amplification

PCR reactions were performed using an ABI Prism 7700 Detection System (Applied Biosystems, Foster City, CA, USA) with a total volume of 25 ml reaction mixture containing 5 ml of each appropriately diluted RT sample (standard curve points and patient samples), 12.5 μl TaqMan Universal PCR Master Mix (Applied Biosystems, Carlsbad, CA, USA), 900 nM of each primer and 250 nM of the probe. The thermal cycling conditions comprised an initial incubation at 50°C for 2 min, a denaturing step at 95°C for 10 min and 40 cycles at 95°C for 15 s and at 65°C for 1 min. Each experiment included a standard curve with five cDNA concentrations, a control sample (OVCAR3 carcinoma cell-line), 25 patients and no template control. The standard curve was generated using serially diluted solutions of standard cDNA derived from the HTB-77 carcinoma cell line. Real-time PCR assays were conducted in triplicates for each sample. Mean ratios of L1CAM/TBP relative quantitations expressed as arbitrary units were used for calculation.

#### Immunohistochemistry technique

L1CAM was determined by immunohistochemistry, as previously described [19, 28]. In brief, 4-μm paraffin sections of each tissue sample were obtained and mounted on Super-frost plus slides. Sections were then deparaffinized in xylene and rehydrated in decreasing grades (100%-70%) of ethanol. Antigen retrieval was done in EDTA 8.0 M in a pressure cooker. The automated IHC procedure was performed with an i6000 BioGenix automatic stainer. Endogenous peroxidase activity was blocked by treating for 10 min with 3% hydrogen peroxide in methanol. To minimize background staining, sections were incubated with normal goat serum. Thereafter, the slides were incubated with anti-L1 (clone 14.10). Immunoperoxidase staining was accomplished with a super-sensitive detection kit. Counterstaining was then performed with hematoxylin.

The samples were analyzed by our pathologist and cases were considered positive when more than 10% of L1CAM-positive cancer cells were present in the stained tissue sections (as previously described [18]).

### Statistical analysis

To evaluate statistical significance of the not normally distributed L1CAM mRNA expression data in relation to the clinicopathological features Wilcoxon-Mann-Whitney test (for two groups) or Kruskal-Wallistest (for more than two groups) was applied. For survival-analysis the L1CAM mRNA-expression was dichotomized into low and high using the median expression value. Univariate survival analysis for overall and progression-free survival was assessed by Kaplan-Meier's method and log-rank test to determine the difference of survival curves. Correlations between factors were analyzed using Pearson correlations. Multivariate analyses were calculated using cox regression model. Results at a level (p-value) of less than 0.05 were considered significant. All statistical analyses were computed using SPSS 21.0.

## SUPPLEMENTARY TABLE




