# Supplementary material for: Clinical impact of L1CAM expression measured on the transcriptome level in ovarian cancer
Source: Oncotarget. 2016 May 11;7(24):37205–14. doi: 10.18632/oncotarget.9291 (PMC5095069; doi:10.18632/oncotarget.9291)
Supplement: Supplementary file 1 [file oncotarget-07-37205-s001.pdf]

Clinical impact of L1CAM expression measured on the transcriptome level in ovarian cancer

SUPPLEMENTARY TABLE

Supplementary Table S1: Overview of all relevant published studies on L1CAM expression and ovarian cancer with the main results.

See Supplementary File 1
