# Supplementary material for: Clinical impact of L1CAM expression measured on the transcriptome level in ovarian cancer
Source: Oncotarget. 2016 May 11;7(24):37205–14. doi: 10.18632/oncotarget.9291 (PMC5095069; doi:10.18632/oncotarget.9291)
Supplement: Supplementary file 2 [file oncotarget-07-37205-s002.docx]

| Author | Number of patients and type of analyzed tissue | Detection Method | Main findings |
| --- | --- | --- | --- |
| Fogel et al 2003 [1] | n=58  (ovarian carcinoma) | IHC (L1-11A and L1-14-10, against the ectodomain of L1) | - 80% of OC L1CAM pos. - L1CAM expression was excellent predictor of poor outlook (p<0.00001) |
| Daponte et al. 2008 [2] | n=81  (cystadenomas n=20,  borderline tumors n=14,  ovarian carcinomas n=47) | IHC, Western plot | - L1CAM immunoreactivity correlated with stage and grade - L1CAM low tumors associated with longer PFS (p=0.002) - L1 CAM expression associated with chemotherapy response |
| Zecchini et al. 2008 [3] | n= 434  (normal ovaries n= 20,  cystadenomas n=4,  epithelial ovarian carcinomas n=211  metastatic lesions n=199) | IHC  (polyclonal antibody pcytL1 - against cytoplasmatic L1 site) | - L1CAM positivity in 100% of normal and 100% benign lesions, in 42.7% of primary EOC and 44.7% of metastases - L1CAM pos. EOC reduced PFS (p=0.03) and OS (p=0.009) |
| Bondong et al. 2012 [4] | n= 232  (serous ovarian carcinomas) | ELISA (mL1CAM in tumor lysates, sL1CAM in ascitic fluid) | - L1CAM-expressing tumors show highly invasive phenotype + associated with restricted resectability at primary debulking surgery + associated with increased lymphogenic spread - Soluble L1CAM is marker for poor PFS and chemoresistance - In ovarian cancer cell lines specific knock- down of L1CAM reduces IL-1β expression and NF-κB activity. |
| Moulla et al. 2013 [5] | n=104  (cyst adenocarcinomas n=29,  borderline ovarian tumors n=50,  carcinomas n=25) | IHC | - L1CAM expression more prominent in borderline and malignant lesion than in benign, no relation with tumor grade or stage found |
| Aktas et al. 2013 [6] | n= 154  (ovarian cancer) | ELISA (in serum) | - Inverse association of L1CAM with platinum sensitivity - prognostic value |

1. Fogel M, Gutwein P, Mechtersheimer S, Riedle S, Stoeck A, Smirnov A, Edler L, Ben-Arie A, Huszar M, Altevogt P: **L1 expression as a predictor of progression and survival in patients with uterine and ovarian carcinomas.** *Lancet* 2003, **362**:869–875.

2. Daponte A, Kostopoulou E, Kollia P, Papamichali R, Vanakara P, Hadjichristodoulou C, Nakou M, Samara S, Koukoulis G, Messinis IE: **L1 (CAM) (CD171) in ovarian serous neoplasms.** *Eur J Gynaecol Oncol* 2008, **29**:26–30.

3. Zecchini S, Bianchi M, Colombo N, Fasani R, Goisis G, Casadio C, Viale G, Liu J, Herlyn M, Godwin AK, Nuciforo PG, Cavallaro U: **The differential role of L1 in ovarian carcinoma and normal ovarian surface epithelium.** *Cancer Res* 2008, **68**:1110–1118.

4. Bondong S, Kiefel H, Hielscher T, Zeimet AG, Zeillinger R, Pils D, Schuster E, Castillo-Tong DC, Cadron I, Vergote I, Braicu I, Sehouli J, Mahner S, Fogel M, Altevogt P: **Prognostic significance of L1CAM in ovarian cancer and its role in constitutive NF-κB activation.** *Ann Oncol* 2012, **23**:1795–1802.

5. Moulla A, Miliaras D, Sioga A, Kaidoglou A, Economou L: **The immunohistochemical expression of CD24 and CD171 adhesion molecules in borderline ovarian tumors.** *Pol J Pathol* 2013, **64**:180–184.

6. Aktas B, Kasimir-Bauer S, Wimberger P, Kimmig R, Heubner M: **Utility of mesothelin, L1CAM and Afamin as biomarkers in primary ovarian cancer.** *Anticancer Res* 2013, **33**:329–336.
